# Characterization of the integrated filamentous phage Pf5 and its involvement in small-colony formation

**DOI:** 10.1099/mic.0.2006/003533-0

**Published:** 2007-06

**Authors:** Marlies J. Mooij, Eliana Drenkard, María A. Llamas, Christina M. J. E. Vandenbroucke-Grauls, Paul H. M. Savelkoul, Frederick M. Ausubel, Wilbert Bitter

**Affiliations:** 1 Department of Medical Microbiology and Infection Control, VU medical centre, van der Boechorststraat 7, 1081 BT Amsterdam, The Netherlands; 2 Department of Genetics, Harvard Medical School and Department of Molecular Biology, Massachusetts General Hospital, Boston, MA 02114, USA

## Abstract

Bacteriophages play an important role in bacterial virulence and phenotypic variation. It has been shown that filamentous bacteriophage Pf4 of *Pseudomonas aeruginosa* strain PAO1 mediates the formation of small-colony variants (SCVs) in biofilms. This morphology type is associated with parameters of poor lung function in cystic fibrosis patients, and SCVs are often more resistant to antibiotics than wild-type cells. *P. aeruginosa* strain PA14 also contains a Pf1-like filamentous prophage, which is designated Pf5, and is highly homologous to Pf4. Since *P. aeruginosa* PA14 produces SCVs very efficiently in biofilms grown in static cultures, the role of Pf5 in SCV formation under these conditions was investigated. The presence of the Pf5 replicative form in total DNA from SCVs and wild-type cells was detected, but it was not possible to detect the Pf5 major coat protein by immunoblot analysis in PA14 SCV cultures. This suggests that the Pf5 filamentous phage is not present at high densities in the PA14 SCVs. Consistent with these results, we were unable to detect *coaB* expression in SCV cultures and SCV colonies. The SCV variants formed under static conditions were not linked to Pf5 phage activity, since Pf5 insertion mutants with decreased or no production of the Pf5 RF produced SCVs as efficiently as the wild-type strain. Finally, analysis of 48 clinical *P. aeruginosa* isolates showed no association between the presence of Pf1-like filamentous phages and the ability to form SCVs under static conditions; this suggests that filamentous phages are generally not involved in the emergence of *P. aeruginosa* SCVs.

## Introduction

*Pseudomonas aeruginosa* is a versatile bacterium that can inhabit many environments, including soil, marshes, plants and water. In its natural environments, *P. aeruginosa* predominantly grows in organized communities called biofilms. Growth as a biofilm is also an important requirement for the colonization of human tissues. For instance, *P. aeruginosa* grows as a biofilm in the lungs of cystic fibrosis (CF) patients, and, despite long-term antibiotic treatment, the lungs remain infected (Hoiby *et al.*, 2001; Singh *et al.*, 2000). Recent work has indicated that phenotypic variants (bacteria that show a stable change in phenotype whether genetic or epigenetic), such as *P. aeruginosa* SCVs, play an important role in biofilm formation (Boles *et al.*, 2004; Kirisits *et al.*, 2005; Webb *et al.*, 2004). Moreover, hyperadherent antibiotic-resistant SCVs also appear after treatment of *P. aeruginosa* PA14 cultures with the antibiotic kanamycin (Drenkard & Ausubel, 2002).

Although the physiology and morphology of bacteria living in biofilm and planktonic cultures appear to be completely different, only 1 % of the genes in the *P. aeruginosa* strain PAO1 genome have been found to be differentially expressed when the two types of growth are compared (Whiteley *et al.*, 2001). The genes that are most highly unregulated during biofilm development (up to 83.5-fold activation) are part of the Pf4 filamentous phage of strain PAO1. Interestingly, Webb *et al.* (2004) have shown that the production of Pf4 phage correlates with the appearance of SCVs in PAO1 mature biofilms. Those authors found phage filaments on the surface of SCVs isolated from biofilms, but not on biofilm cells that had not converted to SCVs. Moreover, wild-type planktonic cells had developed into hyperadherent SCVs after 12 h incubation with purified Pf4 virions. Because it is known that prophages can cause DNA inversions and phenotypic variation (Kutsukake & Iino, 1980; Tominaga, 1997), Webb *et al.* (2004) concluded that Pf4 was a mediator of phase variation in *P. aeruginosa* biofilms. A report by Deziel *et al.* (2001) has also linked the emergence of SCVs in *P. aeruginosa* strain H57RP to phase variation, based on the observation that cells switched at high frequency from the SCV to the wild-type phenotype. However, those authors did not look for the association of a filamentous phage with variant formation (Deziel *et al.*, 2001). Other reports have also described bacteriophages that cause or modulate phase variation. In *Escherichia coli*, filamentous phage f1 infection has been linked to the appearance of small colonies (Kuo *et al.*, 2000), and a recent report by Brockhurst *et al.* (2005) has established that the ssRNA bacteriophage PP7 modulates *P. aeruginosa* colony morphology. Small rough, and large diffuse, colonies have been observed in the presence of PP7 phage, whereas only large diffuse colonies are displayed in its absence (Brockhurst *et al.* 2005).

In contrast to the data reported by Whiteley *et al.* (2001) and Webb *et al.* (2004), suggesting a role of Pf4 in SCV and biofilm formation, transcriptional profiling of SCVs obtained from PAO1 biofilms has shown that Pf4 phage genes are among the most highly downregulated (up to 220-fold reduction with respect to the wild-type) in comparison with planktonic cells (Kirisits *et al.*, 2005). However, the conditions used to grow SCV cultures for transcriptional profiling in the latter study differed considerably from conditions generally used to grow biofilms, making it difficult to directly compare the Whiteley *et al.* (2001) and Kirisits *et al.* (2005) studies. Moreover, it is possible that filamentous phages are important in initiating the formation of SCVs, but not in maintaining the SCV phenotype.

The aim of the present study was to investigate whether Pf1-like bacteriophages are generally involved in small-colony formation in *P. aeruginosa*. To address this question, we focused on the relationship between SCVs produced by *P. aeruginosa* strain PA14, and the presence of a Pf1-like filamentous bacteriophage in the PA14 genome. In addition, we examined the occurrence of Pf1-like bacteriophages in *P. aeruginosa* clinical isolates, and their correlation with SCV appearance.

## Methods

### 

#### Bacterial strains and culture conditions.

Bacterial strains and plasmids used in this study are listed in Table 1. Clinical isolates and primary plates were obtained from the VU medical centre (Amsterdam, The Netherlands); clinical biofilm-related isolates (obtained from urine catheters, and from sputa of CF patients) were obtained from the Public Health Laboratory Friesland (Leeuwarden, The Netherlands), and from the Universite Libre de Bruxelles (Brussels, Belgium). Unique identifiers (mutantID) for all the transposon mutants used in this study are listed in Table 1; further information on these mutants can be found at http://ausubellab.mgh.harvard.edu/cgi-bin/pa14/home.cgi. Batch cultures were routinely grown in liquid Luria–Bertani (LB) medium at 37 °C on a rotary shaker operated at 200 r.p.m., unless stated otherwise. When required, antibiotics were used at a final concentration of: ampicillin, 100 µg ml^−1^; tetracycline, 20 µg ml^−1^; and chloramphenicol, 30 µg ml^−1^. Static culture experiments were performed by inoculating individual colonies in 3 ml LB medium. Cultures were grown vertically in test tubes at 37 °C. At each time point, the top layer of the culture (pellicle) was taken with a swab, and resuspended in PBS solution. Dilution series were plated onto LB agar. The first time point was 3 days. In cases where no SCVs were observed, second and third time points were 4 and 5 days, respectively.

**Table 1.** Bacterial strains and plasmids used in this study

Strain or plasmid	Relevant characteristics	Reference
***E. coli***		
DH5α	*supE44* Δ*lacU169* (ϕ80 *lacZ*ΔM15) *hsdR17 recA1 endA1 gyrA96 thi-1 relA1*	Hanahan (1983)
HB101	*supE44 hsdS20 recA13 ara-14 proA2 lacY1 galK2 rpsL20 xyl-5 mtl-1*	Boyer & Roulland-Dussoix (1969)
***P. aeruginosa***		
PA14	Wild-type	Rahme *et al.* (1995)
PA14_49000	Single transposon insertion in PA14_49000 using the *MAR2xT7*, Gm^r^ (mutantID 28588)	Liberati *et al.* (2006)
PA14_48990	Single transposon insertion in PA14_48990 using the *MAR2xT7*, Gm^r^ (mutantID 24025)	Liberati *et al.* (2006)
PA14_48970	Single transposon insertion in PA14_48970 using the *MAR2xT7*, Gm^r^ (mutantID 25542)	Liberati *et al.* (2006)
PA14_48940	Single transposon insertion in PA14_48940 using the *MAR2xT7*, Gm^r^ (mutantID 25468)	Liberati *et al.* (2006)
PA14_48930	Single transposon insertion in PA14_48930 using the *MAR2xT7*, Gm^r^ (mutantID 36387)	Liberati *et al.* (2006)
PA14_48920	Single transposon insertion in PA14_48920 using the *MAR2xT7*, Gm^r^ (mutantID 25825)	Liberati *et al.* (2006)
PA14_48910	Single transposon insertion in PA14_48910 using the *MAR2xT7*, Gm^r^ (mutantID 33859)	Liberati *et al.* (2006)
PA14_48890	Single transposon insertion in PA14_48890 using the *MAR2xT7*, Gm^r^ (mutantID 41135)	Liberati *et al.* (2006)
PA14_48880	Single transposon insertion in PA14_48880 using the *MAR2xT7*, Gm^r^ (mutantID 37556)	Liberati *et al.* (2006)
**Plasmids**		
pUC19	Cloning vector with *ori*ColE1, *rop* mutant, α-*lacZ* mutant	Lin-Chao *et al.* (1992)
pRK600	Helper plasmid with *ori*ColE1, *mobRK2* and *traRK2*; Cm^r^	de Lorenzo & Timmis (1994)
pMP220	IncP broad-host-range *lacZ* fusion vector; Tc^r^	Spaink *et al.* (1987)
pMPPcoaB	CoaB promoter fragment cloned upstream of *lacZ* gene in pMP220; Tc^r^	This study
pMMB67EH	IncQ broad-host-range plasmid; *lacI*^q^ Ap^r^	Furste *et al.* (1986)
pMMBPf5c	pMMB67EH carrying the *Sac*I–*Xba*I Pf5 fragment subcloned from TOPO II vector in the opposite orientation with respect to P*_tac_*	This study
pMMB717-720	pMMB67EH carrying the *Kpn*I–*Xba*I Pf5 fragment subcloned from pCR II-TOPO in the opposite orientation with respect to P*_tac_* fragment	This study
pMMB727-728	pMMB67EH carrying the *Nhe*I–*Kpn*I Pf5 fragment subcloned from TOPO II vector in the opposite orientation with respect to P*_tac_*	This study
pCoaB Pf4	CoaB Pf4 PCR fragment cloned into pCR II-TOPO upstream of P*_lac_*	This study
pCoaB Pf5	CoaB Pf5 PCR cloned into pUC19 upstream of P*_lac_*	This study

In SCV-induction experiments, strains were grown in LB, CAS (Llamas *et al.*, 2006), and M9 minimal medium (Liberati *et al.*, 2006; Sambrook *et al.*, 1989), with citrate as the carbon source. Late-exponential-phase broth cultures were supplemented with 10mM MgSO_4_.

#### Recombinant DNA techniques.

Recombinant DNA techniques were performed as described by Sambrook *et al.* (1989). Total DNA and plasmid DNA were prepared using Qiagen reagents. Restriction endonucleases and T4 DNA ligase (New England BioLabs) were used according to the manufacturer’s recommendations. PCR was carried out using materials from Applied Biosystems, or using the Expand High Fidelity PCR System (Boehringer Mannheim) for cloning purposes. PCRs were performed using 1 ng template DNA μl^−1^, 200 μM each dNTP, 0.5 μM each primer, and 5 % (v/v) DMSO (final concentration). DNA was extracted from gels using a Qiagen gel extraction kit. Sequencing analysis was performed with the BigDye terminator v3.1 cycle sequencing kit (Applied Biosystems) on an ABI Prism 3100 capillary sequencer (Applied Biosystems), according to the manufacturer’s specifications.

#### Computer-assisted analysis.

The genome of *P. aeruginosa* strain PA14 was analysed for the presence of a Pf4-like phage with kodon version 2.03 (Applied Maths). This program was also used to predict the repeat region of the Pf4-like phage.

#### Western immunoblot analysis.

*P. aeruginosa* wild-type and SCV cells were grown overnight with agitation, and harvested by centrifugation (5 min, 25 000 ***g***). Prior to the centrifugation, SCV cultures were plated to confirm that the bacteria had preserved their phenotype. Bacterial pellets were resuspended in sonication buffer containing 20 mM Tris/HCl (pH 8.0) and 5 mM EDTA. After ultrasonic disruption of the cells, cell envelope proteins were isolated by centrifugation (5 min, 25 000 ***g***). Prior to loading the gel, the samples were incubated for 10 min at 95 °C. Total proteins were subjected to three-layer SDS-PAGE. In short, the stacking gel was 4 % (w/v) acrylamide combined with 10 % (w/v) (approx. 1 cm) and 15 % (w/v) acrylamide separating gels containing 0.13 % (v/v) glycerol. The protein bands were blotted to a Hybond nitrocellulose membrane (Amersham Biosciences), and were probed with either rabbit polyclonal antiserum against phage Pf1 (1 : 250), or pre-immune serum obtained from the same rabbit (Asla Biotech). Two plasmids, one containing the gene encoding the major coat protein (*coaB*) of Pf4, and the other containing *coaB* of Pf5, both under the control of the P*_lac_* promoter, were used as positive controls. *coaB*-Pf4 was amplified with primers CoaB-F 5′-TTTAAGCTTCGTCACTTCTTCGTAAAGCC-3′ (bp 790 948–790 967) and PA724R 5′-GGCCTTGACGCAGGTAGTTC-3′ (bp 792 071–792 052), which were based on the sequence GenBank accession no. AE0040921.2. Subsequently, *coaB*-Pf4 was cloned into a pCR II-TOPO vector (Invitrogen), according to the manufacturer’s recommendations. c*oaB-*Pf5 was amplified with primers CoaB-F 5′-TTTAAGCTTCGTCACTTCTTCGTAAAGCC-3′ (bp 602 035–602 054) and CoaB-R 5′-AAACTGCAGCCAGCTACACAACACCAGC-3′ (bp 602 542–602 524), which were based on the sequence GenBank accession no. NZ_AABQ07000002. The forward primer contains a *Hin*dIII restriction site, and the reverse primer contains a *Pst*I restriction site (restriction sites are underlined in the respective primer sequences). The *Pst*I site was not used in subsequent cloning. The *coaB*-Pf5 PCR product obtained was cloned as a *Hin*dIII–*Eco*RI (the *Eco*RI site was present in the CoaB fragment) fragment into the polylinker of the pUC19 plasmid. *coaB*-Pf4 and *coaB*-Pf5 constructs were transformed into *E. coli* XL10 competent cells, and the presence of *coaB* was confirmed by DNA sequencing. Overnight LB broth cultures of XL10 cells containing the Pf5 or Pf4 *coaB*-expressing plasmids were supplemented with 1 mM IPTG for 3 h prior to protein isolation.

#### *coaB* promoter activity analysis.

Two transcriptional fusions of the region upstream of the major coat protein of phage Pf5 (*coaB*) were made using the pMP220 plasmid, which contains a promoterless *lacZ* gene (Spaink *et al.*, 1987). Specific primers TCoaB-F 5′-AAGAATTCGGCTTGTCAGATTACACTGGG-3′ (bp 599 183–599 203) and TCoaB-R 5′-AATCTAGAGGACATATCACCCTTGCCC-3′ (bp 602 230–602 212) were designed based on the sequence GenBank accession no. NZ_AABQ07000002, and PfTF-F 5′-AAGAATTCGAGACGACGTTGCGATAGG-3′ (bp 789 959–789 977) and PfTF-R 5′-AATCTAGACCACAGTTCGACGACGCC-3′ (bp 790 206–790 190) were designed based on the sequence GenBank accession no. AE004091. Both of the PCR products were cloned as *Eco*RI–*Xba*I fragments (restriction sites are underlined in primer sequences) into the *Eco*RI–*Xba*I sites of pMP220, and transformed into DH5α. The fusion constructs were confirmed by DNA sequencing, and eventually transferred to *P. aeruginosa* PA14 by triparental mating using *E. coli* strain HB101 (pRK600) as the helper strain (de Lorenzo & Timmis, 1994). The SCVs used for this assay were obtained using static culture conditions, and then directly recultured in LB broth with agitation (overnight). Cells were checked for lack of reversion to wild-type phenotype after culturing. β-Galactosidase assay activity from the transcriptional fusion construct was measured as described by Miller (1972). Each assay was run in duplicate at least three times.

#### SCV induction experiments.

Supernatants from late-exponential-phase LB cultures of fresh SCV and wild-type colonies were used to incubate wild-type cells (diluted 300 times), and look for emergence of SCVs. The supernatants were diluted 1 : 2 with fresh medium to supply fresh nutrients to the bacteria, and then filter-sterilized prior to use. All culture incubations were carried out at 37 °C on a rotary shaker operated at 200 r.p.m. Different dilutions of the cultures were plated on LB medium after 1, 3 and 5 days post-incubation. Induction experiments were repeated five times in LB medium, and performed one time each in CAS broth and M9 minimal medium.

#### Detection of the Pf5 replicative form.

To detect the presence of the Pf5 replicative form (RF), and to accurately define Pf5 boundaries within the *P. aeruginosa* PA14 genome, we generated primers designed to amplify the predicted phage recircularization region of the Pf5 RF. Primers Pf5RF-F 5′-ACGGTGGAAACATCCTGGC–3′ (bp 607 816–607 834) and Pf5RF-R 5′-AACAGTGAATTGCGGACAAGG-3′ (bp 597 865–597 845) (GenBank accession no. NZ_AABQ07000002) were used for this purpose. PCR amplifications were performed using total DNA, as well as plasmid DNA (Plasmid Midi kit; Qiagen). The PCR products obtained were cloned into a pCR II-TOPO vector, according to the manufacturer’s recommendations, and they were subsequently sequenced.

#### Complementation analysis.

Pf5 *Mar2xT7* transposon insertion mutants (Table 1) were complemented with a fragment that included the complete Pf5 phage genome, except for the flanking repeats used in RF circularization, and the gene encoding the putative phage regulatory protein (PA14_49030). Primers c-Pf5F 5′-CAACAATTCGACCTATTGCGGG-3′ (bp 599 555–599 576) and c-Pf5R 5′-GCAAAGGAAAAATCTAGGACGTCTCG-3′ (bp 607 989–607 964) (GenBank accession no. NZ_AABQ07000002) were used for this purpose. The PCR reaction was performed using the Extensor Hi-Fidelity PCR master mix (Abgene), according to the manufacturer’s recommendations. The 8.4 kb PCR product was cloned into a pCR II-TOPO vector (pCR II-TOPO Pf5; Invitrogen), and subcloned into the *Sac*I–*Xba*I sites of the broad-host-range plasmid pMMB67EH with the P*_tac_* promoter in the opposite orientation, generating plasmid pMMBpf5c. For the second and third constructs, the 2.8 kb *Kpn*I–*Xba*I fragment and the 2.5 kb *Nhe*I–*Kpn*I fragment of pCR II-TOPO pf5 were subcloned into the *Kpn*I–*Xba*I sites of pMMB67EH, generating pMMB717-720 and pMMB727-728, respectively. The three constructs were transferred from *E. coli* DH5α to *P. aeruginosa* by triparental mating using *E. coli* strain HB101 (pRK600) as a helper strain (de Lorenzo & Timmis, 1994).

#### SCV quantification in Pf5 transposon insertion mutant strains.

Quantification of SCVs in transposon mutants (PA14_49000 : : *Mr2xT7*, PA14_48970 : : *Mr2xT7*, PA14_48890 : : *Mr2xT7* and PA14_48880 : : *Mr2xT7*) was performed after inoculating approximately 2.5×10^8^ bacteria from overnight cultures into glass tubes containing 5 ml fresh LB medium. Then, we incubated the tubes at 37 °C under static conditions for 26 h. At that point, the samples were removed from the incubator, and vortexed vigorously. We then removed 1 ml aliquots from the tubes, and used gentle sonication to dissolve bacterial aggregates completely. Serial dilutions of sonicated cultures were made using PBS, and the dilutions were plated on LB agar plates. SCV and wild-type bacterial colonies were counted using a Leica Wild M3C microscope. We calculated ratios of SCV and wild-type colonies for each dilution plated. Mean values and standard deviations were calculated using data from at least three independent experiments.

#### Prevalence of SCVs and Pf1-like filamentous bacteriophages in *P. aeruginosa* clinical isolates.

Primary plates of 102 *P. aeruginosa* clinical specimens were screened for the presence of SCVs. The presence of the three Pf1-like bacteriophages (Pf1, Pf4 and Pf5) was analysed by performing PCR amplifications in 46 biofilm-related clinical isolates, using primers designed to specifically detect each phage. We also used a universal primer pair designed based on the sequence of a conserved gene that is present in all three phage genomes (designated ORF424 in Pf1, PA0726 in PAO1, and PA14_48910 in Pf5), and which corresponds to a predicted Zot-toxin-like gene. Pf1-specific primers Pf1-F 5′-CTATGAGAATGGTCGTTCCG-3′ (bp 5734–5753) and Pf1-R 5′-CAGAAGATCGACTTGCCC-3′ (bp 6284–6267), Pf4-specific primers Pf4-F 5′-TCGAATTCCGCTTCCATCAC-3′ (bp 795 297–795 317) and Pf4-R 5′-CCTGATGCTTGGTCAGGTACG-3′ (bp 796 297–796 277), and Pf5-specific primers Pf5-F 5′-ATTCACCGAGCTTCGTAGGC-3′ (bp 607 355–607 374) and Pf5-R 5′-GCGGTATCGTATTGCCAAGAG-3′ (bp 607 764–607 744) were designed based on sequences obtained from GenBank (accession nos X52107, NC_002516 and NZ_AABQ07000002, respectively). Universal Pf1-like phage primers pfU-F 5′-GTGTCGATCAAGATCCACCA-3′ (bp 604 150–604 169) and pfU-R 5′-GGAGGAAGAAAGCTATTCGCA-3′ (bp 605 021–605 001) were designed based on the PA14 strain sequence (GenBank accession no. NZ_AABQ07000002). The amplification of the universal Pf1-like phage PCR was combined with the Pf4-specific PCR, in which an annealing temperature of 53 °C was used.

## Results and Discussion

### PA14 Pf1-like filamentous bacteriophage

**Fig. 1.**
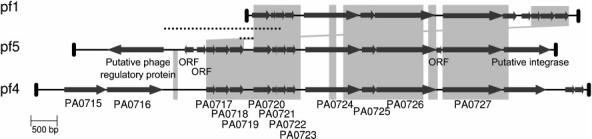
Comparison of the Pf5 genome with the genomes of Pf1 and Pf4. The designations below the Pf4 genome correspond to gene names from the PAO1 genome sequence (Stover *et al.*, 2000). For corresponding designations in Pf1 and PF5, see Hill *et al.* (1991) and Table 2 (this study), respectively. Only additional CDSs present in Pf5 compared with Pf4 are depicted. The black dotted lines indicate regions used to detect the promoter activity of *coaB*.

Genome analysis of *P. aeruginosa* PA14 revealed the presence of a Pf1-like filamentous prophage, which we named Pf5. The genes of Pf5 show conserved synteny and high identity with those of filamentous bacteriophage Pf4 and, to a lesser extent, with those of phage Pf1 (Fig. 1). A major difference between Pf5 and Pf4 is the absence of four Pf4-coding sequences in Pf5 (Table 2). These additional coding sequences (CDSs) encode a putative reverse transcriptase (PA0715), a component of an ABC transporter system (PA0716), a putative prevent-host-death (*phd*) antitoxin protein (intergenic region between PA0728 and PA0729), and a plasmid stabilization toxin protein of the RelE/ParE family (PA0729) (Webb *et al.*, 2004; Table 2). In addition, there are three additional CDSs in Pf5 that encode a putative phage regulatory protein (PA14_49030) and two proteins of unknown function (PA14_49010 and PA14_49020) (Fig. 1, Table 2). Another important difference between Pf4 and Pf5 is the chromosomal location of the integrated bacteriophage genomes. Phage Pf4 is located between PA0714 and PA0730, and Pf5 is inserted between the PA14 homologues of the PAO1 PA1191 (PA14_49040) and PA1192 (PA14_48870) genes. Therefore, genome data show that although Pf1-like bacteriophages are present in both PAO1 and PA14 strains they are fairly diverse, and could play different roles in their respective bacterial hosts. *In silico* analysis of the Pf5 genome showed the presence of a 10 bp direct repeat (TTTGTGCGTA) located in intergenic regions downstream of PA14_49040 (PAO1 PA1191 homologue) and upstream PA14_48870 (PAO1 PA1192 homologue). To detect the presence of the RF of Pf5, and to determine the region of recircularization of the filamentous phage, we designed primers directed toward opposite ends of the putative integrated bacteriophage. A 715 bp DNA fragment was amplified using primers Pf5RF-F and Pf5RF-R from SCVs and wild-type total DNA. Sequence analysis of the fragments obtained showed that phage Pf5 indeed used the 10 bp direct repeats identified by *in silico* analysis to circularize. Based on the flanking regions of the phage genome, the size of the viral genome is 10 675 bp. The size of phage Pf5 is intermediate between the sizes of the viral genomes of Pf1 and Pf4, which are 7349 and 12 437 bp, respectively.

**Table 2.** Name and function of Pf5 genes (strain PA14) and corresponding genes in Pf4 (strain PAO1)

Pf5 (PA14)	Pf4 (PAO1)	Gene product
PA14_49030	No homologue	Putative regulatory protein
PA14_49020	No homologue	Hypothetical
PA14_49010	No homologue	Hypothetical
No homologue	PA0715	Putative reverse transcriptase
No homologue	PA0716	Component of ABC transporter
Pa14_49000	PA0717	Hypothetical
Pa14_48990	PA0718	Hypothetical
Pa14_48980	PA0719	Hypothetical
Pa14_48970	PA0720	Helix-destabilizing protein
Pa14_48960	PA0721	Hypothetical
Pa14_48950	PA0722	Hypothetical
Pa14_48940	PA0723	Coat protein B
Pa14_48930	PA0724	Putative coat protein A
Pa14_48920	PA0725	Hypothetical
Pa14_48910	PA0726	Hypothetical
Pa14_48890	PA0727	Hypothetical
Pa14_48880	PA0728	Probable bacteriophage integrase
No homologue	Intergenic region between PA0728 and PA0729	Putative prevent-host-death (*phd*) antitoxin protein
No homologue	PA0729	Plasmid stabilization toxin protein

### Emergence of SCVs in PA14

**Fig. 2.**
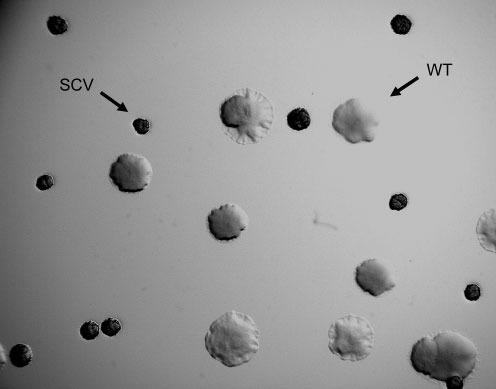
Wild-type (WT) colonies and SCVs derived from a 3-day-old static liquid culture of PA14.

The filamentous bacteriophage Pf4 has been associated with the formation of SCVs in *P. aeruginosa* strain PAO1 (Webb *et al.*, 2004). Therefore, we studied the effect of Pf5 on SCV formation in *P. aeruginosa* PA14. First, the ability of PA14 to form SCVs on biofilms formed on the air–liquid interface of cultures incubated under static conditions was studied. After 3 days' incubation, approximately 70 % of all colonies in the air–liquid interface biofilm showed an SCV phenotype (Fig. 2). Subsequently, we tested whether incubation of wild-type PA14 bacteria with PA14 SCV supernatants (which potentially contain the Pf5 phage) was able to induce the emergence of SCVs, as shown for PAO1 by Webb *et al.* (2004). Although approximately 10 % of wild-type cells were converted to SCVs by the addition of SCV supernatants, this effect was not specific for the supernatant of SCVs, since identical results were obtained when the supernatant of wild-type cells was used. Moreover, PA14 SCVs did not contain superinfective phages able to form lytic plaques on PA14 wild-type bacteria (data not shown), unlike the SCVs described by Webb *et al.* (2004).

### Analysis of Pf5 phage production in PA14 wild-type and SCV bacteria

**Fig. 3.**
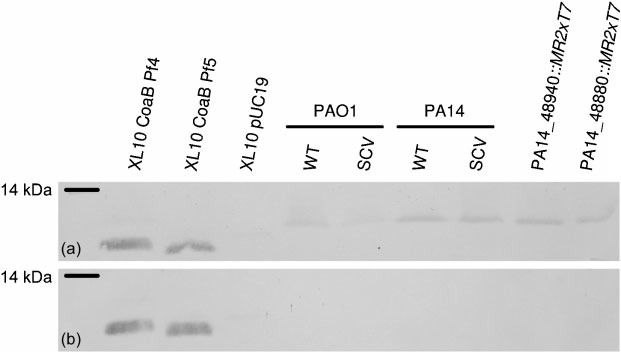
Immunoblot analysis using Pf1 antiserum. (a) Cell envelope proteins (b) Secreted proteins. PA14_48940 : : MR2xT7 is a *coaB* mutant, and PA14_48880 : : MR2xT7 is a RF mutant. Total protein from XL10 *E. coli* cells, complemented with different constructs, was used as a control in (a) and (b).

Although we detected the production of the RF of Pf5 by PCR, we found no indication that Pf5 phage was responsible for the emergence of the SCVs under static conditions. To determine whether Pf5 phages were produced by SCVs, we used a polyclonal antibody directed against filamentous phage Pf1 in immunoblot experiments. In addition, we also determined whether Pf4 phages were produced by wild-type cells and SCVs of strain PAO1. Because the antibody used in our experiments was directed against Pf1, we tested the specificity of the Pf1 antiserum against Pf4 and Pf5 coat protein B by cloning and expressing the Pf4 and Pf5 *coaB* genes in *E. coli*. We were able to detect a protein band of 8 kDa (corresponding to the size of the CoaB protein) using both constructs when we used the Pf1 antiserum, but not when we used the pre-immune serum (Fig. 3). In immunoblots performed using cultures from PA14 SCVs and wild-type bacteria (see Methods), we were unable to detect the Pf5 CoaB protein (Fig. 3). In addition, cultures from PAO1 wild-type cells and PAO1 SCVs obtained using static conditions were also tested in immunoblots using Pf1 antiserum, and, again, we were unable to detect the major coat Pf4 protein on PAO1 samples. This suggests that the CoaB protein is not produced at detectable levels by either wild-type cells or static SVCs in either the PAO1 or the PA14 strain (Fig. 3). Results obtained with two PA14 mutants that contain transposon insertions in *coaB* (PA14_48940) and the putative bacteriophage integrase (PA14_48880, which does not produce the Pf5 replicative form) showed that the high-molecular-mass band present in the Western blot was due to a non-specific reaction with the Pf1 antiserum (Fig. 3). The results obtained with the PAO1 strain were somewhat unexpected, since Webb *et al.* (2004) showed expression of Pf4 phage protein in PAO1 SCVs obtained from biofilms. Therefore, our results suggest that formation of small colonies in *P. aeruginosa* is likely to involve other mechanisms in addition to the production of filamentous bacteriophages.

To confirm that the CoaB protein is not produced at detectable levels in PA14 bacteria, we analysed the promoter activity of the gene encoding the major coat protein (*coaB*) of Pf5 in both wild-type cells and SCVs. We cloned two different PCR fragments, both of which contained the putative promoter region of PA14 *coaB* (Fig. 1, dotted lines) upstream of a promoterless *lacZ* reporter gene of pMP220, and introduced the construct in *P. aeruginosa* PA14. The results obtained showed no expression of *coaB* in either the SCVs or the wild-type cells of PA14, suggesting that Pf5 *coaB* is not expressed at detectable levels. As a control, we plated SCV colonies containing the *lacZ* reporter directly on medium supplemented with X-Gal. None of the SCVs plated showed observable β-galactosidase activity. Together, these results show that *coaB* is not expressed above background levels in *P. aeruginosa* PA14, independent of the colony phenotype.

### Analysis of phage Pf5 mutant strains

**Fig. 4.**
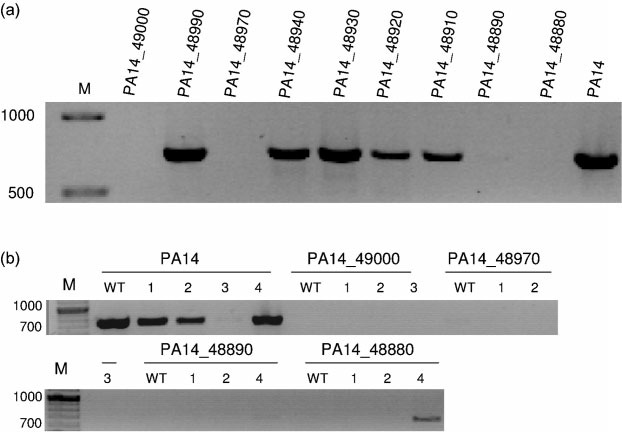
Detection of the Pf5 RF by agar gel electrophoresis. (a) PCR products obtained from Pf5 transposon mutants. (b) Complementation of Pf5 transposon mutants showing decreased RF production with plasmids pMMB67EH (1), pMMBpf5c (2), pMMB717-720 (3) and pMMB727-728 (4). Marker sizes (M) are shown in base pairs.

The results presented so far suggest that Pf5 is not involved in the emergence of PA14 SCVs in biofilms gown under static conditions. In order to show this unequivocally, we analysed nine transposon insertion mutants in the Pf5 region (Table 1). We first extracted total DNA from the transposon mutants, and looked for the presence of the Pf5 RF by PCR, using primers Pf5RF-F and Pf5RF-R. As shown in Fig. 4(a), four transposon insertion mutants (PA14_49000 : : *Mr2xT7*, PA14_48970 : : *Mr2xT7*, PA14_48890 : : *Mr2xT7* and PA14_48880 : : *Mr2xT7*) consistently showed no PCR product corresponding to the Pf5 RF. Two of the mutants that showed absence of the RF PCR product have transposon insertions in genes involved in bacteriophage replication. Mutant PA14_48970 : : *Mr2xT7* contains the insertion in a gene encoding a Pf1 single-stranded DNA-binding protein homologue responsible for the formation of a pre-assembly complex with the viral DNA, and the insertion in mutant PA14_48880 : : *Mr2xT7* is located in a gene encoding a putative phage integrase (PA14_48880). We were unable to detect the major coat protein in cultures from the mutant PA14_48880 : : *Mr2xT7* using Pf1 antiserum (Fig. 3). The other two mutants contain insertions in the PA14_49000 and PA14_48890 CDSs that encode two hypothetical proteins of 70 and 432 aa, respectively. A blast search of the proteins encoded by these genes showed a high degree of homology to *Pseudomonas* Pf1 and Pf4 proteins (Hill *et al.*, 1991; Webb *et al.*, 2004). Moreover, the protein encoded by PA14_48890 also showed 46 % similarity to a replication initiation factor from *Geobacter metallireducens*.

In order to confirm that the genes PA14_49000, PA14_48970, PA14_48890 and PA14_48880 are involved in the production of the RF, we tested whether plasmid pMMBpf5c, which contains most of the Pf5 genome (except for the repeat regions and the putative phage regulator gene PA14_49030), could complement the four insertion mutants. However, this plasmid did not complement the RF phenotype in any of the mutants (Fig. 4b). The expression of the Pf5 genes from a medium-copy-number plasmid could have a negative effect on the production of the RF, although this was not evident when pMMBpf5c was introduced into the wild-type strain. We made two new constructs with shorter regions of the Pf5 genome, and used them to complement the mutants. One of these plasmids (pMMB727-728) contained the PA14_48890 and PA14_48880 genes, and the other (pMMB717-720) contained a total of 12 genes, including genes PA14_48940 (*coaB*)–PA14_49050 (downstream of the PA14_49030 phage regulator). As shown in Fig. 4(b), mutant PA14_48880 (putative integrase) could be complemented with plasmid pMMB727-728, showing that PA14_48880 is indeed involved in RF production. Complementation of PA14_48890 with the same plasmid (pMMB727-728) was less clear, as only very small amounts of the RF were detected by PCR (Fig. 4b). Expression of plasmid pMMB717-720 in PA14 wild-type prevented RF production (Fig. 4b), and this indicates that this construct contains a gene that represses RF production. Not surprisingly, plasmid pMMB717-720 did not complement mutants PA14_49000 : : *Mr2xT7* and PA14_48970 : : *Mr2xT7* (Fig. 4b). Since the four genes identified in Pf5 that are potentially involved in RF production have homologues in PAO1 Pf4, it would be interesting to test PAO1 mutants in those particular genes, in order to confirm the involvement of Pf4 phage in SCV formation in strain PAO1.

Subsequently, we examined the ability of the mutants that failed to produce Pf5 RF (PA14_49000 : : *Mr2xT7*, PA14_48970 : : *Mr2xT7*, PA14_48890 : : *Mr2xT7* and PA14_48880 : : *Mr2xT7*) to generate SCVs under static conditions. Results obtained on the emergence of PA14 SCVs under static conditions have shown that SCVs can be consistently quantified when culture media are inoculated with known numbers of wild-type bacteria (E. Drenkard and F. M. Ausubel, unpublished data). Mutant analysis performed using the Pf5 transposon insertion mutants showed no significant differences in the number of SCVs that emerged at the end of the incubation period (26 h) between any of the mutants tested and the wild-type strain. Moreover, no morphological differences were observed when comparing SCV colonies formed by the mutants and wild-type bacteria. These results unambiguously demonstrate that, unlike what was reported previously for strain PAO1, there is no link between emergence of SCVs and Pf1-like phage activity in *P. aeruginosa* strain PA14 under the conditions used.

### Prevalence of SCVs and Pf1-like bacteriophages in clinical specimens

Pf1-like filamentous phages have now been found in three *P. aeruginosa* isolates (Hill *et al.*, 1991; Webb *et al.*, 2004). To determine the prevalence of SCVs in clinical specimens, 102 primary plates containing *P. aeruginosa* colony isolates obtained directly from patient samples (i.e. blood, sputum, urine, and wound material) were analysed for SCVs. Only two of the primary plates analysed showed colonies with an SCV phenotype (ear infection, and sputum from a CF patient). These data show that SCVs are not very common among clinical samples, but their presence may be more common in certain infection sites, such as the respiratory tract of CF patients, that provide a niche for highly adherent *P. aeruginosa* SCVs (Häussler *et al.*, 2003). Although the prevalence of isolates producing SCVs is low, it does not rule out the possibility that these strains are capable of forming small colonies. Therefore, we checked 48 clinical biofilm-related isolates, i.e. *P. aeruginosa* isolated from urine catheters and sputa of CF patients, for their ability to form SCVs. Upon static culturing of these *P. aeruginosa* isolates, 27 % formed SCVs (Table 3), and this indicates that a reasonable number of *P. aeruginosa* clinical isolates have the ability to form SCVs under these conditions. Because some *P. aeruginosa* strains, such as strain PAO1, form SCVs at very low frequencies under static conditions (0.024 % of PAO1 colonies were SCVs after 65 h incubation, compared with 76 % for strain PA14), it is possible that we have underestimated the number of clinical isolates able to form SCVs in the present study. Subsequently, we tested the 48 biofilm-related isolates for the presence of the three known Pf1-like filamentous bacteriophages (Pf1, Pf4 and Pf5) by PCR amplification. First, the quality of the genomic DNA isolated from the different isolates was tested using PCR primers that target the *P. aeruginosa* PAO1 housekeeping gene PA2968 (*fabD*), which was detected in all clinical isolates (data not shown). Subsequently, we performed PCR reactions using primer pairs specific for each of the three *Pseudomonas* phages, and one PCR reaction that targeted all three phages by using a universal primer pair (see Methods) that was designed based on the sequence of a CDS common to Pf1, Pf4 and Pf5 (PA14_48910). Phage Pf1 ORF100 and ORF90 (Hill *et al.*, 1991) were used as the targets for the Pf1-specific PCR, PA0727 and PA0728 (Webb *et al.*, 2004) were use for the Pf4-specific PCR, and the putative integrase gene of Pf5 (PA14_48880) was used for the Pf5-specific PCR. These experiments showed that six isolates showed PCR amplification with the Pf4-specific primers, two isolates were positive in the Pf5-specific PCR, and none were positive in the Pf1-specific PCR (Table 3). The universal Pf1-like primers amplified specific PCR products from 26 isolates, and five of these were positive for either Pf4 or Pf5 (Table 3). This shows that, although filamentous phages are common among clinical *P. aeruginosa* isolates, they seem to be very diverse. This result is in agreement with whole-genome DNA microarray data obtained by Wolfgang *et al.* (2003), who showed that among 18 clinical and environmental *P. aeruginosa* strains, different isolates contain different subsets of the filamentous phage Pf4 genes. Comparison of the PCR data with the ability to form SCVs showed that only 9 of the 29 clinical isolates that had amplification products for at least one of the filamentous phages tested produced SCVs (Table 3). These results suggest that filamentous phages are generally not involved in the emergence of *P. aeruginosa* SCVs, and this is consistent with the data obtained using the PA14 strain

**Table 3.** Association between the presence of Pf-like genes, as detected by PCR, and the ability to form SCVs in biofilm-related clinical isolates

SCV phenotype	Pf1-specific	Pf4-specific	Pf5-specific	Only Pf universal*	No Pf product	Total
Positive	0	2 [2]†	1 [1]†	6	4	13 (27 %)
Negative	0	4 [2]†	1 [0]†	15	15	35 (73 %)
Total	0 (0 %)	6 (12 %)	2 (4 %)	21 (44 %)	19 (40 %)	48

*Positive in the Pf-universal PCR only.

†Values in square brackets indicate the number of isolates that were also positive for the Pf-universal PCR.

In summary, this study identified a Pf1-like filamentous bacteriophage in the genome of *P. aeruginosa* strain PA14, and it was named Pf5. Since strain PA14 forms SCVs very efficiently, we investigated the involvement of the Pf5 filamentous phage in SCV formation. We amplified a fragment that corresponds to the RF of Pf5 using DNA preparations from SCVs and wild-type cells, but we did not observe production of phage virions under the assay conditions used. Additionally, we were unable to detect the major coat protein of phage Pf4 and Pf5 (CoaB) by Western immunoblot analysis using PAO1 and PA14 SCV cultures, suggesting that the filamentous phages are not present in high amounts in SCVs from strain PAO1 and PA14. Consistent with these results, measurements of activity of the *coaB* gene promoter in strain PA14 did not detect levels of *coaB* expression in SCV cultures and SCV colonies. Finally, we showed that Pf5-insertion-mutant strains with decreased or no production of the Pf5 RF produced SCVs as efficiently as the wild-type strain, clearly demonstrating that variant formation under static conditions is not linked to Pf5 phage activity in *P. aeruginosa* strain PA14. Results obtained using 48 clinical biofilm-related isolates suggest that the presence of bacteriophages of the Pf family does not correlate with the ability of the bacterial host to form SCVs under static conditions in *P. aeruginosa*.
